# Willingness to Participate in Health Research: Mexican and Mexican American Women's Perspectives

**DOI:** 10.1089/whr.2022.0036

**Published:** 2022-09-20

**Authors:** Yareli Cornejo-Torres, Emily Boniface, Edlyn Lopez, Katherin Gomez-Arboleda, Blair G. Darney

**Affiliations:** ^1^ Department of Obstetrics and Gynecology, Oregon Health and Science University, Portland, Oregon, USA.; ^2^ Portland State University, Portland, Oregon, USA.; ^3^ Department Health Systems and Policy, OHSU-PSU School of Public Health, Portland, Oregon, USA.; ^4^ Instituto Nacional de Salud Pública (INSP), Centro de Investigación en Salud Poblacional (CISP), Cuernavaca, Morelos, México.

**Keywords:** health research, Mexican, Mexican American, recruitment, research participation

## Abstract

**Background:**

Lack of racial and ethnic diversity in health research negatively impacts generalizability. We describe Mexican and Mexican American women's willingness to participate in health research in Oregon.

**Methods:**

We conducted a survey with Mexican-origin Latinas aged 18–49 years. Our primary outcome was willingness to participate in health research; we also asked sociodemographics and barriers and facilitators to participation. We used logistic regression to identify factors associated with willingness to participate.

**Results:**

Of 500 participants, 41% said that they would be willing to participate in health research, 14% said no, and 45% were unsure. In multivariable analyses, past participation in research and speaking English well were independently associated with willingness to participate. Barriers to participation included language, accessibility, and fear of medical procedures. Facilitators included improving future health care, language, and free medical care.

**Conclusions:**

Mexican-origin Latinas in Oregon are willing to participate in health research, but many are unsure. Providing study materials in Spanish is a concrete first step to improve recruitment and promote equity and inclusion.

## Introduction

Despite the National Institutes of Health (NIH)^1^ and Food and Drug Administration^2^ efforts to increase the inclusion of underrepresented communities in clinical trials, in the United States, overall 81% of clinical trial participants are white.^3,4^ This lack of representation from non-white racial and ethnic groups has a negative impact on the generalizability of health research in the United States.^5^ Lack of representation has negative impacts for both the efficacy and effectiveness of treatments and interventions. For example, the efficacy of treatments for conditions that disproportionately affect non-white individuals is threatened if non-white individuals are not included in trials.

In addition, the effectiveness of interventions that require patient engagement and compliance to succeed can be jeopardized if underrepresented patients are not included.^5,6^ Lack of inclusion in health research also negatively impacts equity and inclusion and can foster increased distrust of health research and health care.^7,8^ Evidence about challenges to African American, Native American, and Asian American^9^ participation in health research exists. Commonly cited barriers are mistrust in doctors and the health system based on historical and current examples of discrimination and harm, mistrust, and language barriers.^10,11^ Less is known about barriers to participation in health research by Latinx/o/a or Hispanic individuals and communities.

Close to one in five (18%) of the nation's population identifies as Latinx, the second largest ethnic group in the country.^12^ However, <8% of participants in the NIH clinical trials are Latinx,^13^ and a recent review of cancer clinical trials reported that only between 2% and 5% of the participants identified as Latinx. Increasing the representation of Latinx individuals in health research studies is recognized as key to improving preventive care, as well as understanding why some conditions such as diabetes disproportionately impact the Latinx population.^14^

In Oregon, Latinx people make up 12% of the population,^15^ and the vast majority are of Mexican origin. Nearly one quarter (23%) of births in Oregon are to Latina women.^15^ Our academic medical center prioritizes increasing racial and ethnic diversity in research studies. The medical center undertakes a variety of clinical research studies with reproductive-aged women. However, the medical center study participants are overwhelmingly non-Hispanic white women. The purpose of this study was to describe Mexican and Mexican American women's willingness to participate in health research and identify barriers and facilitators to participation to inform strategies to improve recruitment of Latinas in women's health research.

## Methods

We conducted a cross-sectional survey study. Our bicultural, bilingual (English and Spanish) Mexican-origin and Colombian-origin research team members recruited 500 Spanish- or English-speaking Latinas between the ages of 18–49 years who are waiting for processes and services (such as passports, legal assistance, and notary appointments) at the Consulate General of Mexico in Portland, Oregon. The Consulate serves both Mexican-born and U.S.-born individuals of Mexican origin; U.S.-born individuals of Mexican origin can, for example, apply for dual citizenship or request Mexican passports or birth certificates from the Consulate.

We recruited a convenience sample between November 2019 and February 2020. Respondents who met the screening criteria (self-identified Latina, 18–49 years old) provided oral informed consent after reviewing an information sheet and chose their preferred language for the self-administered survey. Participants completed the survey on paper; responses were then entered into REDCap (Research Electronic Data Capture^16^) electronic database by trained study staff. The senior author resolved any questions about survey responses. The Oregon Health and Science University Institutional Review Board approved this study.

We developed our survey based on the previous literature^17^ and our own formative research with stakeholder partners. The Consulate General of Mexico participated in the development and translation of the survey to ensure that our questions and wording were culturally appropriate in Spanish and English. We then piloted the survey with local Latinas of diverse education levels. Finally, we worked with a colleague at the National Institute of Public Health in Cuernavaca, Mexico, to do a final check for grammar and spelling. Our survey included 22 questions and included sociodemographics, health care utilization, and whether participants had heard of health research, willingness to participate in health research in the future, and barriers and facilitators to participation.

Our primary outcome was willingness to participate in health research (yes/no/I do not know). We collapsed the outcome to a binary variable (yes vs. no/I do not know) for multivariable modeling. Our secondary outcomes were knowledge of health research at our medical center, and facilitators and barriers to participation in health research. We assessed seven potential facilitators: free medical care (annual examination or women's health examination), free birth control, incentive (money) given for participation, personal interest in the research topic, possibility of improving health care for other Latinas in the future, possibility of seeing a flyer or advertisement about the study in Spanish, and possibility of having information about the study presented in Spanish.

We assessed seven potential barriers as well: mistrust in medical providers (doctors) and hospitals or Universities, accessibility of location, finding childcare, language barriers, fear of medical procedures, not understanding what it means to participate in health research, and hearing bad things from other individuals and/or health research study participants. Respondents were also able to submit open-ended responses; we analyzed these thematically and reclassified them to the existing facilitators and barriers where possible.

We examined eight sociodemographic independent variables: survey language, age, living outside the Portland metropolitan area, years in the United States, relationship status, education level, employment type, and self-rated ability to speak English. We categorized age into four groups: 18–24, 25–34, 35–44, and 45 years or older for descriptive analyses, and collapsed these into two groups for regression modeling: 18–34 and 35 years or older. We classified years in the United States into four groups: 0–10, 10–15, >15, and all of my life, collapsing 0–10 and 10–15 into a single group for regression modeling. We grouped the highest education level completed as less than high school/General Educational Development (GED), high school/GED, and at least some college. We classified primary sources of employment into three groups: private company or government, self-employed or family business/farm, and work at home or unemployed. Finally, we categorized self-reported ability to speak English as not at all, somewhat, or very well.

We also assessed six independent variables focused on health systems utilization and experience: health insurance status, primary source of medical care, having seen a doctor in the last year, needing an interpreter at the doctor, past participation in health research, and past knowledge of our academic medical center. We categorized primary sources of medical care into four groups: hospital or clinic, urgent care or emergency room, community clinic or health fair, and other sources. If a respondent selected both urgent care/emergency room and another medical care source, we prioritized the other source, as we were most interested in investigating non-emergency sources of primary health care. We examined all “other” responses and reclassified them into the offered categories, if applicable. We classified insurance status as insured or uninsured/unknown, and all other variables as binary (yes or no).

Our unit of analysis is the survey respondent. We characterized both sociodemographic information and health systems utilization of respondents overall and by willingness to participate in health research using Pearson's chi-squared tests or Fisher's exact tests. We described the proportion of respondents who selected each facilitator and barrier to health research participation, excluding missing responses. We used logistic regression to assess characteristics associated with willingness to participate in health research, in both bivariate and multivariable models. We performed a sensitivity analysis to test the robustness of our multivariable regression model by including both education level and ability to speak English. We hypothesized that the addition of time in the United States might affect the model, but its inclusion did not meaningfully alter any associations, so we excluded this variable from final models. We used Stata version 15.1 for all analyses.

## Results

Our survey sample included 500 Latina respondents, of whom 456 (91.2%) took the survey in Spanish (Table 1). The majority of respondents lived in the greater Portland metropolitan area (54.0%), had lived in the United States for >15 years (53.0%), and did not have a relationship partner (74.6%). Respondents were most likely to be between the ages of 35 and 44 years (41.4%), to have completed less than high school or a GED (40.6%), to be employed by a private company or the government (40.4%), and reported that they spoke English somewhat (44.4%).

**Table 1.** Demographics of Study Participants, by Potential Participation in Future Research Study (*n* = 500)

Characteristic	Overall (***n*** = 500)	Yes (***n*** = 185)	No (***n*** = 62)	Don't know (***n*** = 199)	***p***
Survey language					0.141
English	44 (8.8)	23 (12.4)	6 (9.7)	13 (6.5)	
Spanish	456 (91.2)	162 (87.6)	56 (90.3)	186 (93.5)	
Age, years					0.299
18–24	58 (11.6)	24 (13.0)	5 (8.1)	27 (13.6)	
25–34	126 (25.2)	54 (29.2)	21 (33.9)	47 (23.6)	
35–44	207 (41.4)	77 (41.6)	20 (32.3)	81 (40.7)	
≥45	107 (21.4)	30 (16.2)	16 (25.8)	44 (22.1)	
Missing	2 (0.4)	0 (0)	0 (0)	0 (0)	
Live in greater Portland metropolitan area					0.160
No	218 (43.6)	74 (40.0)	31 (50.0)	89 (44.7)	
Yes	270 (54.0)	108 (58.4)	28 (45.2)	108 (54.3)	
Missing	12 (2.4)	3 (1.6)	3 (4.8)	2 (1.0)	
Years in the United States					0.298
0–10	70 (14.0)	28 (15.1)	8 (12.9)	29 (14.6)	
10–15	74 (14.8)	21 (11.4)	7 (11.3)	37 (18.6)	
>15	265 (53.0)	101 (54.6)	31 (50.0)	102 (51.3)	
All my life	85 (17.0)	35 (18.9)	15 (24.2)	29 (14.6)	
Missing	6 (1.2)	0 (0)	1 (1.6)	2 (1.0)	
Relationship status					0.084
Partnered	122 (24.4)	53 (28.7)	10 (16.1)	51 (25.6)	
Not partnered	373 (74.6)	129 (69.7)	52 (83.9)	148 (74.4)	
Missing	5 (1.0)	3 (1.6)	0 (0)	0 (0)	
Education					0.194
Less than high school/GED	203 (40.6)	65 (35.1)	23 (37.1)	92 (46.2)	
High school/GED	147 (29.4)	56 (30.3)	20 (32.3)	58 (29.2)	
At least some college	111 (22.2)	55 (29.7)	15 (24.2)	37 (18.6)	
Missing	39 (7.8)	9 (4.9)	4 (6.4)	12 (6.0)	
Employment type					0.646
Private company/government	202 (40.4)	74 (40.0)	31 (50.0)	85 (42.7)	
Self-employed/family business	64 (12.8)	29 (15.7)	8 (12.9)	23 (11.6)	
At home/unemployed	173 (34.6)	66 (35.7)	16 (25.8)	71 (35.7)	
Missing	61 (12.2)	16 (8.7)	7 (11.3)	20 (10.1)	
How well do you speak English					0.179
Very well	151 (30.2)	69 (37.3)	21 (33.9)	57 (28.6)	
Somewhat	222 (44.4)	83 (44.9)	22 (35.5)	92 (46.2)	
Not at all	104 (20.8)	30 (16.2)	16 (25.8)	45 (22.6)	
Missing	23 (4.6)	3 (1.6)	3 (4.8)	5 (2.5)	

Data are expressed as *n* (%).

GED, General Educational Development.

**FIG. 1.**
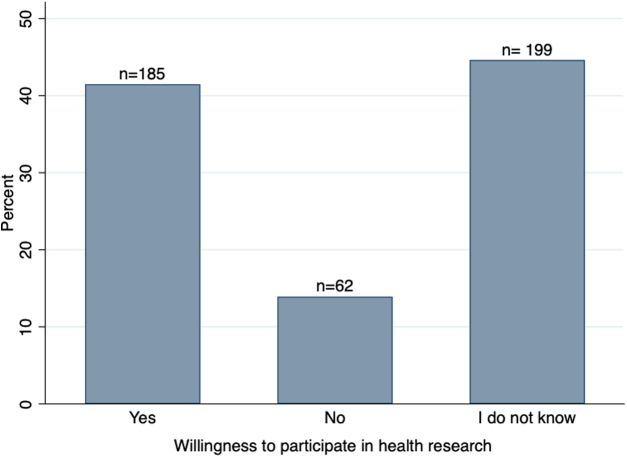
Willingness of survey respondents to participate in health research. Bar heights represent the percentage of non-missing responses; counts of responses are indicated at the top of each bar (*n* = 446). Fifty-four respondents were missing responses.

Of the 500 respondents, 446 respondents answered the primary research question, and of these, 41.5% (185/446) were willing to participate in health research, 13.9% (62/446) were not willing, and 44.6% (199/446) did not know (Fig. 1). Latinas who were willing to participate in health research tended to be younger, have a relationship partner, have completed a higher level of education, and to have higher self-reported English ability than Latinas who said that they were not willing to participate or did not know. However, none of these differences were statistically significant (Table 1).

Half of our sample (49.6%) had health insurance, and almost 70% had seen a doctor in the last year (Table 2). Respondents sought medical care at hospitals/clinics and community clinics/health fairs in approximately equal numbers (41.6% and 45.8%, respectively), with only 5.2% using urgent care/emergency rooms as their primary care source. More than half (53.2%) of respondents required an interpreter when visiting the doctor. Large majorities of the sample had not previously participated in health research (88.0%) or heard of our academic medical center (78.4%).

**Table 2.** Health Systems Utilization and Experience, by Willingness to Participate in Future Research Study (*n* = 500)

Characteristic	Overall (***n*** = 500)	Yes (***n*** = 185)	No (***n*** = 62)	Don't know (***n*** = 199)	***p***
Health insurance					
Insured	248 (49.6)	98 (53.0)	34 (54.8)	98 (49.3)	0.631
Uninsured/don't know	222 (44.4)	83 (44.9)	25 (40.3)	92 (46.2)	
Missing	30 (6.0)	4 (2.2)	3 (4.8)	9 (4.5)	
Where do you go for medical care?					0.313
Hospital or clinic	208 (41.6)	82 (44.3)	28 (45.2)	82 (41.2)	
Urgent care/ER	26 (5.2)	8 (4.3)	2 (3.2)	11 (5.5)	
Community clinic/health fair	229 (45.8)	89 (48.1)	25 (40.3)	89 (44.7)	
Other	14 (2.8)	3 (1.6)	4 (6.5)	6 (3.0)	
Missing	23 (4.6)	3 (1.6)	3 (4.8)	11 (5.5)	
Seen doctor in last year					0.230
No	147 (29.4)	47 (25.4)	19 (30.7)	64 (32.2)	
Yes	348 (69.6)	136 (73.5)	42 (67.7)	135 (67.8)	
Missing	5 (1.0)	2 (1.1)	1 (1.6)	0 (0)	
Needed interpreter at doctor					0.196
No	219 (43.8)	94 (50.8)	28 (45.2)	82 (41.2)	
Yes	266 (53.2)	88 (47.6)	31 (50.0)	113 (56.8)	
Missing	15 (3.0)	3 (1.6)	3 (4.8)	4 (2.0)	
Ever participated in health research?					0.325
No	440 (88.0)	160 (86.5)	57 (91.9)	180 (90.5)	
Yes	33 (6.6)	18 (9.7)	2 (3.2)	10 (5.0)	
Missing	27 (5.4)	7 (3.8)	3 (4.8)	9 (4.5)	
Did you know OHSU does women's health research studies before reading about it above?					0.031
No	392 (78.4)	143 (77.3)	45 (72.6)	163 (81.9)	
Yes	87 (17.4)	40 (21.6)	12 (19.4)	30 (15.1)	
Missing	21 (4.2)	2 (1.1)	5 (8.1)	6 (3.0)	

Data are expressed as *n* (%).

ER, emergency room; OHSU, Oregon Health and Science University.

There were almost no statistically significant differences between Latinas who were willing to participate in health research and those who were not or were unsure, although those who expressed willingness tended to be more likely to have seen a doctor in the last year, have participated in past health research, and have no need for an interpreter compared with other respondents. Prior knowledge of our academic medical center was the only variable that differed significantly by our primary outcome: Latinas who were aware of the research program were more likely to be willing to participate in health research (21.6%) than those that were not or who were unsure (19.4% and 15.1%, respectively; *p* = 0.031).

**FIG. 2.**
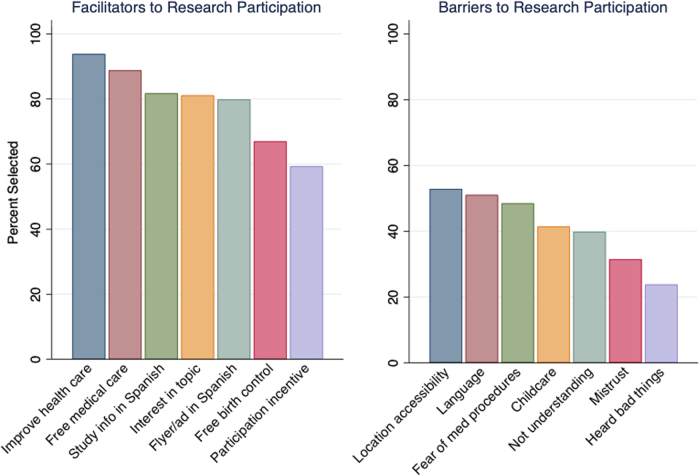
Percentage of participants who selected each barrier and facilitator to future participation in research, excluding missing responses.

Larger proportions of respondents identified facilitators to research participation than barriers (Fig. 2). Among facilitators, the possibility of improving health care for Latinas in the future and free medical care were the most popular responses (93.9% and 88.8%, respectively), while the possibility of having study information in Spanish, study advertisements in Spanish, and interest in the research topic were all selected by ∼80% of respondents. Incentive for participation was selected by the smallest proportion of respondents (59.4%). Among barriers, location of accessibility, language barriers, and fear of medical procedures were the most common responses (52.9%, 51.1%, and 48.5%, respectively). Having heard bad things from other individuals and/or health research study participants was the least common response (23.8%).

In open-ended responses, participants described facilitators and barriers in their own words; we present exemplar quotes.

- “Es bueno participar nada mas que a veces es por falta de tiempo. Y el lugar en donde se tiene que asistir.” (“It is good to participate, just sometimes it's the lack of time and also the location.”)
- “Me gustaría mas información de como son las investigaciones si no son peligrosas para mi salud.” (“I would like more information about what health research is and if it would or would not be dangerous to my health.”)

**FIG. 3.**
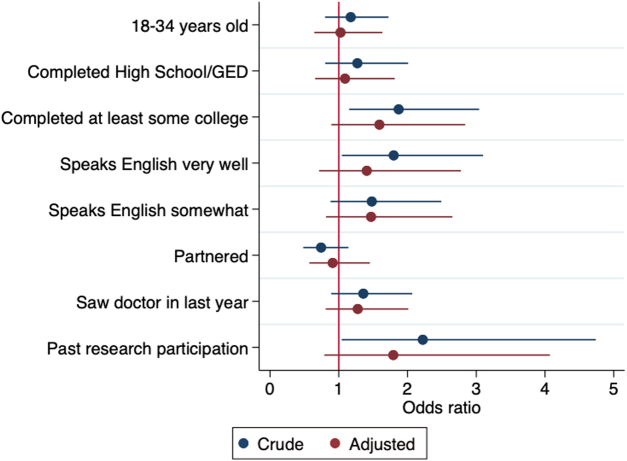
Logistic regression modeling results for primary research question “Would you consider participating in a health research study?” Outcome is answer of yes versus no/do not know (*n* = 400). GED, General Educational Development.

In bivariate logistic regression analyses, multiple variables were significantly associated with increased odds of being willing to participate in health research (Table 3; Fig. 3). Latinas who had completed at least some college had higher odds than those who had not completed high school or a GED (odds ratio [OR] = 1.87, 95% confidence interval [CI]: 1.15–3.04), Latinas who spoke English very well had higher odds than those who did not speak English at all (OR = 1.80, 95% CI: 1.04–3.10), and Latinas who had participated in health research were positively associated with willingness to participate in health research. However, in our multivariable model, these variables lost statistical significance, although the direction of associations persisted, suggesting that these factors may be important, even when controlling for other factors (Table 3; Fig. 3).

**Table 3.** Logistic Regression Modeling Results for Primary Research Question “Would You Consider Participating in a Health Research Study?”

Variable	Level	Crude			Adjusted		
		OR	95% CI	***p***	OR	95% CI	***p***
Age	35 Years or more	Referent	—	—	Referent	—	—
	18–34 Years	1.17	0.80–1.72	0.414	1.03	0.64–1.63	0.915
Education level completed	Less than high school/GED	Referent	—	—	Referent	—	—
	High school/GED	1.27	0.80–2.01	0.307	1.09	0.66–1.81	0.738
	At least some college	1.87	1.15–3.04	0.012	1.59	0.89–2.83	0.116
How well do you speak English?	Not at all	Referent	—	—	Referent	—	—
	Somewhat	1.48	0.88–2.49	0.140	1.41	0.71–2.78	0.324
	Very well	1.80	1.04–3.10	0.034	1.47	0.81–2.65	0.201
Relationship status	No partner	Referent	—	—	Referent	—	—
	Partnered	0.74	0.48–1.14	0.174	0.91	0.57–1.45	0.696
Seen doctor in last 12 months	No	Referent	—	—	Referent	—	—
	Yes	1.36	0.89–2.07	0.156	1.28	0.81–2.01	0.294
Past research participation	No	Referent	—	—	Referent	—	—
	Yes	2.22	1.04–4.74	0.039	1.79	0.79–4.07	0.163

Outcome is answer of yes versus no/do not know (*n* = 400).

CI, confidence interval; OR, odds ratio.

## Discussion

In our sample of Mexican-origin Latinas (Mexican and Mexican American) in Oregon, the majority were willing to participate in health research (41.5%) or are not sure (44.6%). Only 13.9% said that they would not participate. We also identified common barriers to participation: language, accessibility (time and location), and fear of medical procedures. Facilitators most commonly cited by participants were the opportunity to improve medical care for others in the future, language, and receiving free medical care. Past participation in research and speaking English well were independently associated with willingness to participate in health research in the multivariable model.

Previous research on willingness of non-white women to participate in research has also reported high level of willingness to participate. For example, a study of African American women's perspectives on participation in breast cancer clinical trials found that 80% were willing to participate.^18^ However, we found that the largest proportion of our sample (47%) was not sure about participating, which suggests that the Latina population may or lack knowledge about what health research is about or what participation involves, which has also been found in previous research.^19^ It may also suggest a lack of trust in medical personnel, which has been documented previously in black and Native American populations^8^ and was cited as a barrier by participants in our study.

The most common barriers endorsed by our participants were: language, accessibility (time and location), and fear of medical procedures. Our study participants cited language as both a barrier and a facilitator, highlighting its importance. The need to have research study materials and advertisements available in Spanish is vital to increasing participation in Latinx population. It is also important to remember that not all Mexicans speak and read Spanish; additional languages may be necessary (*e.g.*, Nahuatl, Zapotec). Accessibility, time, and mistrust or fear of deportation^10^ have been cited as barriers to participation in research by people of color in previous studies. Fear of medical procedures, a key barrier in our study, may be linked with mistrust. The long history of discriminatory practices such as forced sterilizations^20,21^ and harms in clinical trials for black and brown communities^21–23^ is ongoing and contribute to fear and mistrust of the health care system and health care providers.

A study looking at barriers to clinical trial participation among the Mexican American community found that not speaking English, time, and transportations were identified as barriers to participation,^24^ similar to our findings. In addition, another qualitative study shared that fears related to experimentation, harm, immigration status, and lack of opportunities to participate are all barriers^25^ for the Latinx population. We did not assess immigration status in our study, but we did note that ability to speak English, a common measure of acculturation,^26^ was associated with willingness to participate in research.

We found that the most commonly cited facilitator to participation in health research was to improve health care for others in the future. Other work with Latinx and African American communities found that a desire to help others is a motivator for participation in research.^14,27^ Additional facilitators in our study were language and receiving free medical care. The Latinx population remains the most likely population to be uninsured,^28^ and health research can offer access to needed services, although is not a solution to equitable access to care.

Based on these study findings, we have several recommendations to improve recruitment of Latinas into health research. All study materials must be available in Spanish, and bilingual study staff must be available. Face-to-face recruitment, which was key to the success of this survey study, may also foster trust and improve recruitment.^29^ Location and time of study participation must be more accessible, for example, outside of work hours, providing transportation or childcare, and having signage in Spanish to locate the research office.

We have begun to implement these changes in our own academic medical center. For example, we have begun to use our local Spanish radio station to advertise studies and we have seen an increase in interest in participation among Spanish-speaking women. Finally, researchers must consistently collect and report race and ethnicity data; a study of two decades of registered clinical trials found that among trials that reported any race and ethnicity enrollment data, Latino/a ethnicity was less likely to be reported than other groups (*i.e.*, than Black, Asian, American Indian, and Alaska Native).^4^

Our findings must be interpreted with the following limitations in mind. We recruited at the Mexican Consulate, and thus, our results may not be generalizable to all Latinas in Oregon. We are likely underpowered for some associations, based on our large CIs in our multivariable model; however, directions of bivariate associations were robust to multivariable examination. We did not directly ask about immigration status as a potential barrier in our survey, based on the recommendation of our community partners. However, we did ask about language and years in the United States, which are important measures of acculturation. We have missing data for barriers and facilitators and are not able to extrapolate to respondents who did not answer these items. However, those participants missing facilitators and barriers information were not more likely to say that they would not participate in health research than those who provided information about barriers and facilitators.

## Implications for Practice and Policy

Research participation among Latinas of Mexican origin (Mexicans and Mexican Americans) is a scientific and ethical imperative to improve generalizability of research findings, foster greater trust in health care,^30^ and promote equity and inclusion. Researchers can make participation more accessible by advocating for multiple languages to be available on all study materials in the study planning phase.^5^ In addition to providing materials in Spanish, researchers can facilitate participation by ensuring Spanish-speaking research staff are available,^5^ maintaining accessible hours, and providing transportation or childcare.

Since the completion of this study, the Women's Health Research Unit at our academic medical center has implemented bilingual study materials and patient facing platforms such as recruitment, consents, information sheets, social media^31^ and web page,^32^ and patient communication messages. Interpreter scheduling is now implemented as part of the study setup if needed. We have also worked to make study participant compensation more equitable, recognizing that not all study participants have a Social Security Number. The Women's Health Research Unit is an example of a research space that is actively working on making an inclusive space for our Mexican and Mexican American women in Oregon.

## Conclusions

Our study suggests that Latinas of Mexican origin in Oregon are willing to participate in health research or are unsure. Targeted outreach can focus on key facilitators—for example, being able to improve care for others in the future—and address barriers—for example, language, accessibility (time and location), and fear of medical procedures.

## Abbreviations Used

CI: confidence interval
ER: emergency room
NIH: National Institutes of Health
OHSU: Oregon Health and Science University
OR: odds ratio
